# Antifungal Activity of Two Root Canal Sealers against Different Strains of *Candida*

**DOI:** 10.22037/iej.2017.20

**Published:** 2017

**Authors:** Farnaz Jafari, Sanaz Jafari, Hossein Samadi Kafil, Tahereh Momeni, Helen Jamloo

**Affiliations:** a *Department of Endodontics, Dental School, Tabriz University of Medical Sciences, Tabriz, Iran; *; b *Department of Orthodontics, Dental School, Ilam University of Medical Sciences, Ilam, Iran; *; c *Drug Applied Research Center, Tabriz University of Medical Sciences, Tabriz, Iran; *; d *Dentist, Dental School, Tabriz University of Medical Sciences, Tabriz, Iran*

**Keywords:** Antifungal, Antimicrobial, Candida, Endodontic Sealers

## Abstract

**Introduction:**

Microorganisms and microbial products are the main etiologic factors in pulp and periapical diseases. The present study aimed to compare the antifungal activity of two different sealers, AH-26 and MTA Fillapex against three strains of *Candida,* 24, 48, 72 h and 7 days after mixing.

**Methods and Materials:**

The microorganisms used in this study were* Candidia albicans (*ATCC 10231)*, Candidia glabrata* (ATCC 90030) and *Candidia krusei* (DSM 70079). This test was based on growth of microorganisms and turbidity measurement technique using a spectrophotometer. The direct contact test was conducted by direct and indirect methods. Multiple comparisons were carried out using analysis of variances (ANOVA) with repeated measures followed by Tukey’s tests.

**Results:**

The antifungal activity of both sealers was similar in the indirect method. The antifungal activity of both sealers in the direct method was similar against *Candida albicans *and higher for AH-26 sealer against *Candida krusei* and* Candida glabrata*.

**Conclusion:**

The total antifungal effect of MTA Fillapex sealer was significantly less than AH-26 sealer in direct method. The antifungal effect of both sealers was similar in indirect method.

## Introduction

Microorganisms and microbial products are the main etiologic factors in pulp disease and periapical lesions [1]. Therefore, the main goal in endodontic treatment is to remove pathogens from infected root canals [2]. In order to achieve this goal, cleaning of the canal should be done through instrumentation, and the canal should be obturated with materials owning antimicrobial properties [3, 4].

The most common fungi in endodontic infections is *Candida* genus. The most common strains of the *Candida* are found in oral infections including *Candida albicans *(*C. albicans*) and *Candida glabrata* (*C. glabrata*) as well as *Candida krusei (C. krusei) *[5, 6]. In most studies, *Candida* strains were occasionally detected in primarily infected root canals [-].

Although a molecular analysis detected *C. albicans* in 21% of primary endodontic infections [11], Nair *et al. *[12] introduced this strain as a resistant strain to endodontic treatment. *C. albicans* is also predominant in persistent or refractory periapical lesions [13, 14]. It has a known affinity to dentine and absolute sensitivity to chlorhexidine [15] and partial resistant to calcium hydroxide [15, 16]. Therefore, the antifungal effect of sealer in reducing the number of microorganisms that are left in root canal as well as preventing them from re-growing after root canal treatment is important.

Gutta percha and different sealers are commonly used to obturate root canals. MTA Fillapex (Angelus, Londrina, PR, Brazil) is a new sealer with mineral trioxide aggregate (MTA) base [17]. The philosophy behind the production of this sealer was the presence of MTA in its composition which has many applications in endodontic practice [18]. The pH of this sealer was approximately estimated to be 10-10.5 during setting [17]. However, this new sealer lacks comprehensive scientific information about it [17].

The properties of MTA included in this sealer are alkaline and antimicrobial properties (21). The limited studies previously carried out on antifungal properties of AH-26 [19] and MTA-based sealers [-] were done mainly by agar diffusion test and on *C. albicans* microorganism. Contrary to the agar diffusion test (ADT), the direct contact test (DCT) can show the antimicrobial activity of insoluble components [17].

The purpose of the present study was to compare the antifungal effect of these two sealers on *C. albicans*, *C. glabrata* and *C. krusei* using direct contact test method at 24, 48, 72 h and 7 days after mixing.

## Materials and Methods

In this study, the antifungal properties of two commonly used sealers in the market including MTA Fillapex (Angelus, Londrina, PR, Brazil) and resin-based AH-26 (Dentsply, De Trey, Konstanz, Germany) were studied using direct contact test *in vitro*.

In this study, *C. albicans* (ATCC 10231), *C. glabrata* (ATCC 90030) and *C. krusei* (DSM 70079) were used to evaluate the antifungal properties of materials in root canal therapy. 

All the strains used were standard fungal strains. The stocks were stored at -70^°^C and were recultivated in blood agar culture medium, so that fresh cultivation was used for the study. All biological samples were cultivated for 48 h in Ambient Tryptic Soy Broth (TSB) (Difco Laboratories, Detroit, Mich., USA) before they were used in the study. Direct contact test with both direct and indirect contact of fungal species were performed.

***Direct contact test***

This test was done based on a turbidity survey of microorganisms’ growth in microtiter plates with 96 wells. Schematically, three flat microtiter plates with 96 wells were chosen and classified into different wells to examine the antifungal effect of sealers at different times. Categorization was done at different time intervals. Both sealers were mixed according to manufacturers’ instructions, allowed to set and sterilized with ethylene oxide gas.

In the direct method, the side walls of wells were smeared evenly to 25 mL of the studied sealer so that the material was not transferred to the end of the well because it prevents the measurement of turbidity after cultivation and gives false positive results. Thereafter, 10 µL of bacterial suspension (suspension of each fungus individually containing physiological saline solution and medium containing fungi) with concentration of 10^6^ was coated on the surface of the sealers and placed in the well. The plates were kept vertically, sealed and incubated in 37^°^C to evaporate the liquid containing fungus and to ensure direct contact between fungi and sealer. Thereafter, 245 mL of liquid medium TSB was added and gently shaken for 2 min (Direct Method). In the indirect method, 15 mL of medium was taken and was transferred to another well containing 215 mL of fresh medium. The stated protocol obtained from Anumula [23].

As a result, the growth of fungi in direct contact with the sealer and without sealer (indirect) was studied. Plates were incubated at 37^°^C for 24 h and were read at 600 nm to detect changes in fungal growth. The experiments were repeated in triples for each well in order to ensure accuracy of results. Results of the experiments were recorded based on turbidity creation. Turbidity assessed visually and by a spectrophotometer device (BioTeck, Winooski, VT, USA). This test was repeated at the time rate of 24, 48, 72 h and 7 days after mixing of both sealers.

***Data analysis***

Data was evaluated using descriptive statistics (mean±SD) and ANOVA repeated measures followed by Tukey’s post hoc test to compare antifungal activity of both sealers at each time, using statistical SPSS software (SPSS version 20.0, SPSS, Chicago, IL, USA). In this study, *P*-value less than 0.05 was considered statistically significant.

## Results

The mean and standard deviation of values obtained from the spectrophotometer on the impact of MTA Fillapex and AH-26 sealers on various microorganisms by direct and indirect method were shown in Tables 1 and 2. Comparison of antifungal features of both sealers is shown in Table 1. Spectrophotometric numbers were normalized by subtracting free cell numbers for all wells. In positive control wells containing microorganism without sealers complete microorganism growth was observed.

In direct method (Table 2), for MTA Fillapex sealer, the lowest antifungal activity was on *C. krusei* and the highest activity was on *C. albicans*. In AH-26 sealer group, the lowest antifungal activity was on *C. albicans *and the highest activity was on *C. glabrata.* Also, against *C. albicans*, two sealers had similar activity. Against* C. krusei* and *C. glabrata*, antifungal activity of MTA Fillapex sealer was significantly lower than AH-26. The antifungal activity of MTA Fillapex against *C. glabrata* was not affected by contact time.

In indirect method (Table 2), antifungal activity of MTA Fillapex and AH-26 sealers on all studied strains were similar. In all three Candida genus *C. glabrata*, *C. krusei* and *C. albicans*, two sealers had similar antifungal activity, in indirect method.

## Discussion

The purpose of root canal treatment is to remove the bacteria and fungi from the root canal system and create a proper environment for healing. Complete removal of microorganisms is impossible even by cleaning, shaping and rinsing with antimicrobial substances. Therefore, the use of obturating materials with antimicrobial properties is considered to be helpful in achieving this aim [1, 2].

In the present study, blank cell spectrophotometric numbers were reduced from all wells for normalization. Antifungal features of MTA Fillapex sealer in direct method were time dependent and decreased over time. In indirect method, antifungal activity of this sealer was not time dependent. In addition, the antifungal activity of both sealers in the direct method was more than that in the indirect method.

Little data have been published on antifungal properties of MTA Fillapex sealer. Ozcan *et al. *[21], stated that, 7-day antifungal activity of set MTA Fillapex against *C. albicans *(using agar diffusion method) was not affected by time and this result was not consistent with the results of this study because of the conflict in the type of test method. Due to the low wettability of sealer [24] and the effect of sealer wettability on ADT’s results, this conclusion was justified.

The present findings in 24 h and direct method were consistent with Madani *et al.* [22] who stated that the antifungal features of MTA Fillapex against *C. albicans within *24 h was more than AH-26. Historically, two different assays have been applied to test the antimicrobial characteristics of endodontic sealers, the direct contact test (DCT) and agar diffusion test (ADT). In the present investigation, DCT was used. In the ADT, the results of test depends on its wettability and infusibility in culture medium. The DCT, in contrary, is independent of the solubility and diffusion of the test material [24], and is performed to test set sealers [17]. 

**Table 1.** Mean (SD) of antifungal activity of AH-26 and MTA Fillapex sealers against three Candida species during different intervals using the direct and indirect techniques

			**24 h**	**48 h**	**72 h**	**7 day**	***P *** **value** *
**Direct**	***C. Albicans ***	**AH-26**	1.547^a^(0.091)	0.921^b^(0.018)	0.781^b^(0.069)	0.643^b^(0.021)	0.000
		**MTA**	1.027^a^(0.064)	0.800^b ^(0.053)	0.76^b^(0.029)	0.660^b^ (0.05)	0.000
		***P*** value**	0.001	0.657	0.412	0.322	
	***C. Krusei ***	**AH-26**	1.005^a ^(0.137)	0.542^b^(0.037)	0.515^b^(0.097)	0.491^b^(0.012)	0.000
		**MTA**	2.620^a^(0.192)	2.438^ab^(0.108)	2.33^ab^(0.208)	2.10^b^(0.173)	0.037
		***P*** value**	0.000	0.000	0.000	0.000	
	***C. Glabrata ***	**AH-26**	0.8747^a^(0.089)	0.567^b^(0.69)	0.537^b^(0.530)	0.508^ b^(0.119)	0.000
		**MTA**	1.893(0.357)	1.826(0.337)	1.833(0.270)	1.831(0.212)	0.991
		***P*** value**	0.003	0.003	0.001	0.002	
**Indirect**	***C. Albicans ***	**AH-26**	2.33^a^(0.065)	2.157^b^(0.084)	2.046^b^(0.091)	2.00^b^(0.021)	0.032
		**MTA**	2.56^a^(0.034)	2.34^a^(0.023)	1.970^b^(0.026)	1.977^b^ (0.013)	0.000
		***P*** value**	0.19	0.02	0.29	0.13	
	***C. Krusei ***	**AH-26**	2.436^a^(0.030)	2.153^b^(0.115)	2.126^b^(0.098)	1.965^c^(0.097)	0.000
		**MTA**	2.308^a ^(0.023)	2.203^ab^(0.108)	1.98^b^(0.084)	2.013^b^(0.025)	0.000
		***P*** value**	0.26	0.08	0.06	0.47	
	***C. Glabrata ***	**AH-26**	2.29^a^(0.063)	2.209^ab^(0.026)	2.144^b^(0.037)	2.103^b^(0.010)	0.002
		**MTA**	2.236^a^(0.026)	2.151^ab^(0.106)	2.111^ab^(0.077)	2.008^b^(0.048)	0.008
		***P*** value**	0.24	0.14	0.62	0.03	

* *P value* from repeated measures ANOVA to compare the antifungal effects of sealers over time;

** *P value* from t-test to compare the two sealer types

**Table 2.** The mean (SD) of antifungal activity of MTA Fillapex and AH-26 sealers on three *Candida* species using direct and indirect techniques

		**MTA Fillapex**	**AH-26**	***P value*** **
**Direct**	***C. albicans ***	0.815^c^(0.144)	0.974^a^(0.363)	0.172
	***C. krusei ***	2.372^a^(0.246)	0.638^b^(0.233)	0.000
	***C. glabrata ***	1.845^b^(0.257)	0.622 ^b^(0.162)	0.000
	***P value*** *	0.000	0.004	
**Indirect**	***C. albicans ***	2.215(0.286)	2.135(0.145)	0.398
	***C. krusei ***	2.123(0.146)	2.146(0.144)	0.708
	***C. glabrata ***	2.129(0.097)	2.188(0.081)	0.122
	***P*** value*	0.441	0.561	

* *P value* from repeated measures ANOVA to compare the antifungal effects of sealers over time;

** *P value* from t-test to compare the two sealer types

**Figure 1:**
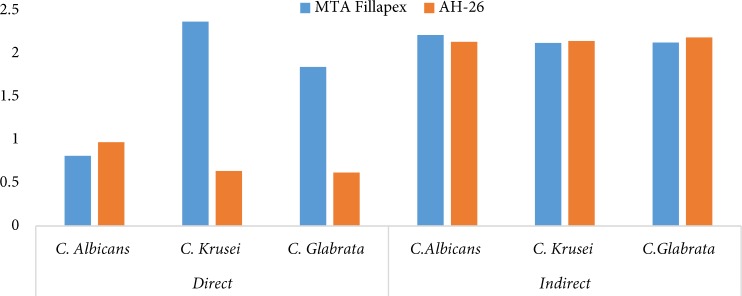
Comparison of antifungal activity of MTA Fillapex and AH-26 sealers on three Candida species using the direct and indirect techniques

In this study, it was observed that in the direct method MTA Fillapex had less antifungal features on *C. krusei* and *C. glabrata*, than AH-26. In this study, the results of antifungal features of AH-26 sealer obtained in direct method was effective in the antifungal features of AH-26 for indirect methods in all studied microorganisms except for *C. albicans* which was effective over time. Also, *C. krusei* and *C. glabrata*, are rare endodontic pathogens [5] and as obtaining microorganism-free canal is the important factor for endodontic success, studying antifungal features of all endodontic materials, even against rare pathogens, can be an important feature. 

In this study, the results of antifungal features in MTA Fillapex sealer showed that in the direct method except for *C. glabrata*, time was effective in the antifungal feature of sealer and in indirect method; time was effective in antifungal features of MTA Fillapex sealer and all microorganisms. In addition, antifungal effect of sealer in the direct method was more than that in the indirect method. This result is consistent with previous studies on antibacterial features of these sealers [25].

Meanwhile, the results of the present study represented that in direct method for MTA Fillapex, the lowest and highest antifungal activity was on *C. krusei* and *C. albicans, *respectively.This result consists with the results of another study by authors about antibacterial activity of AH-26 and MTA Fillapex sealers. For AH-26, and in direct method, the lowest antifungal activity was observed on *C. albicans* and the highest activity was on *C. glabrata*. In addition, in indirect method for all three fungi, *C. glabrata*, *C. krusei*, and *C. albicans* for both sealers, antifungal activity was observed similar.

Putting together the results of direct and indirect methods of direct contact test, AH-26 was preferred over MTA Fillapex according to its antifungal activity.

## Conclusion

Comparison of the two MTA Fillapex and AH-26 sealers showed that in the direct method, antifungal activity of MTA Fillapex sealer was significantly less than AH-26. In the indirect method, antifungal activity of both sealers was similar.
